# Supplementary material for: Are carbon emissions trading and green financial instruments synergistic? -Comprehensive quantitative research based on content analysis
Source: PLoS One. 2024 Mar 7;19(3):e0298601. doi: 10.1371/journal.pone.0298601 (PMC10919601; doi:10.1371/journal.pone.0298601)
Supplement: S1 Data — (ZIP) [file pone.0298601.s001.zip › Data/地级市Z_F工作报告.zip!/╔╜╢1⁄2/╚╒╒╒╩╨/└┤╫╘╣1⁄2╓┌║┼í╛┬φ┐╦╩2╛▌═°í┐_2011.docx]

各位代表：

　　现在，我代表市人民政府向大会作政府工作报告，请予审议，并请市政协各位委员提出意见。

　　一、“十一五”政府工作回顾

　　“十一五”时期是我市在攻坚克难中奋进、在彰显特色中跨越、在抢抓机遇中提升的五年。五年来，在省委、省政府和市委的正确领导下，在市人大、市政协的监督支持下，市政府团结依靠全市人民，认真落实科学发展观，深入实施“四大战略”和“双轮驱动”，加快构建现代产业、民生保障、公共安全、生态环境“四大体系”，积极推进转方式、调结构，有效应对国际金融危机、节能减排等方面的困难与挑战，全力推动科学发展、跨越发展、和谐发展，圆满完成了“十一五”规划确定的主要目标任务，经济社会发展迈上新台阶。

　　这是全市经济快速攀升，综合实力实现新跨越的五年。初步测算，全市生产总值突破千亿元大关，从2005年的424亿元增加到2010年的1030亿元，年均增长15.1%；人均生产总值达到37000元，是2005年的2.4倍。境内财政总收入从86.4亿元增加到349.9亿元；地方财政收入突破50亿元大关、达到55.6亿元，是2005年的3.8倍，年均增长30.4%。规模以上工业增加值突破600亿元，是2005年的3.7倍，年均增长26.3%。港口货物吞吐量从8421万吨增加到2.27亿吨，集装箱吞吐量突破100万标箱，实现了历史性跨越；全市进出口总值达到133亿美元，跨入全省第一方阵，跻身全国百强市行列。五年累计完成固定资产投资2511亿元，是“十五”时期的3.5倍。社会消费品零售总额突破300亿元，是2005年的2.4倍。银行业金融机构本外币存款余额突破1000亿元，信用总量扩大到1440亿元，分别是“十五”末的2.9倍和3.5倍。游客接待量突破2000万人次，旅游总收入突破120亿元，分别是2005年的2.6倍和3.1倍。我们可以自豪地说，过去五年我市发展跃上了新的台阶，今天的日照站在了新的历史起点上！

　　这是结构调整深入推进，优势主导产业迅速崛起的五年。三次产业结构由16.4∶47.2∶36.4调整为9.7∶56.3∶34，地方财政收入占GDP的比重从3.5%提高到5.4%。农业结构调整深入推进，综合生产能力不断提升。粮食总产连续六年稳定在100万吨以上；茶叶、水产、林果、瓜菜、桑蚕、黄烟、畜禽等优质高效农业加快发展，绿茶产量占到全省的70%以上，成为名副其实的北方第一茶乡。以水利化、机械化为重点的农业装备水平明显提升，农田有效灌溉面积超过170万亩，主要农作物耕种收机械化水平超过70%。民营经济和县域经济加快发展，有9个乡镇财政收入过亿元。临港产业迅猛发展，新型工业化进程明显加快。主营业务收入过亿元的企业发展到315家，其中过百亿元的两家；利税过千万元的达到310家。高新技术产业产值达到490亿元，是“十五”末的11倍，占规模以上工业总产值的比重提高13个百分点。新兴汽车产业基地初具规模，现代威亚发动机产能达到40万台，现代派沃泰自动变速箱项目开工建设，五征集团新增汽车产能15万辆，龙泰集团30万台微型乘用车项目开工。黄海粮油二期120万吨粮油加工项目、亚太森博浆纸二期100万吨木浆项目、华能电厂二期2×680兆瓦发电项目建成投产。尤其令人振奋的是，日照钢铁精品基地被列入国家钢铁产业调整振兴规划和全国钢铁产业结构调整试点，一个具有世界先进水平的现代化大型钢铁基地将在日照崛起。

　　服务业增加值达到350亿元，是“十五”末的2.3倍，港口物流、金融保险等现代服务业蓬勃兴起，成为推动全市经济发展的新兴力量。先进制造业、高新技术产业、临港产业集群和现代服务业的发展表明，我市产业结构正在加速优化，转方式、调结构迈出了坚实的步伐！

　　这是基础设施水平全面提升，城乡面貌显著变化的五年。城市规划体系日臻完善，编制完成了城市发展战略规划和土地利用总体规划，开展了新一轮城市总体规划修编。城市载体服务功能全面提升，生态宜居特色更加鲜明。五年累计完成市政建设投资199亿元，建成了奥林匹克水上公园、阳光海岸梦幻海滩、游泳馆等一批标志性工程，水上运动基地被评为“新中国成立60周年百项经典暨精品工程”；新增城市道路450万平方米，建成区面积扩大到89.8平方公里，绿化覆盖率达到43%。新一轮港口规划获得批复，五年累计完成港口建设投资131亿元，是“十五”期间的1.9倍，港口生产性泊位增加到49个，总通过能力超过1.5亿吨。“十二五”建设日照机场的争取工作取得突破性进展。新菏兖日铁路电气化改造工程送电通车，山西中南部至日照港铁路大通道开工建设。胶州至日照天然气长输管道贯通运营。建成了日照水库向市区供水三期和“北水南调”二期工程，供水能力比“十五”末翻了一番。农村基础设施建设实现新突破，在全省率先实现全部行政村通硬化路；全面完成了8座大中型水库、400余座小型水库的除险加固；农村自来水普及率由49%提高到91%；建成农村社区服务中心96处、综合超市332个，农村现代经营服务体系覆盖面达到95%，社会主义新农村更加欣欣向荣。城乡面貌的新变化，使我市“联合国人居奖”的成果得到巩固和提升，人们生活和发展的环境更加美好、更加和谐。

　　这是对外开放上档升级，各项改革深入推进的五年。日照经济开发区晋升为国家级开发区、日照保税物流中心(B型)封关运行、岚山国家级木材贸易加工示范区获准设立，鲁南(日照)国际贸易与航运服务中心建成启用，清大华创孵化器被批准为国家级孵化器，对外开放的载体建设实现新突破。招商引资成效明显，五年累计招引亿元以上的项目252个，到位资金1056亿元，实际利用外资16.3亿美元，在我市投资的世界500强企业由3家增加到18家。接轨青岛全面深化，与半岛城市群、鲁南经济带和沿亚欧大陆桥地区的合作取得新成果。企业改革不断深化，农村综合改革、医药卫生体制改革顺利推进，新一轮政府机构改革基本完成。鲁南临港产业区区域开放合作综合配套改革试点方案获省政府批复进入实施阶段。企业上市取得新突破，日照港、恒宝食品成功上市，累计融资48.6亿元，后备上市资源梯次逐步形成。

这是城乡居民生活水平明显提高，社会事业全面进步的五年。各级政府保障和改善民生的力度进一步加大，五年来，财政用于保障和改善民生的投入达162亿元，年均递增42.4%，完成市级为民办实事重点项目62件。五年累计新增城镇就业22.4万人，转移农村劳动力14.8万人，城镇登记失业率控制在3.2%以内。城镇基本医疗保险和新型农村合作医疗保险覆盖面分别达到98.7%和97.7%。东港、岚山、五莲和日照开发区新型农村社会养老保险纳入国家试点，莒县进入省级试点。农村“五保”对象集中供养率达到71%。城乡居民收入稳步提高，生活条件明显改善。城镇居民人均可支配收入和农民人均纯收入分别达到17500元和7300元，年均增长12.3%和12.1%。启动实施了38个城中村的改造工程，城乡居民人均住房面积分别达到30.3平方米和39平方米，家庭汽车、移动电话、互联网普及率显著提高。科教文卫体等社会事业全面发展。五年取得重要科技成果541项，争创中国名牌产品3个、中国驰名商标2个。全面实现城乡免费义务教育，改制高中全部恢复公办性质，高中段教育普及率达到91%。大学科技园在园学校达到8所，在校生6万人。完成了19处乡镇卫生院、960处村卫生室的业务用房改造和基本医疗设备配备。实现了全部乡镇有综合文化站、全部行政村有文化大院或文化活动室，有线电视实现“村村通”，新编大型历史剧《毋忘在莒》成功晋京演出。我市运动员在第十六届亚运会上勇夺1金1银1铜，在帆板世界杯赛上夺得冠军，实现了亚运会和帆板世界杯赛金牌零的突破。认真落实计划生育、环境保护、土地利用基本国策，在省人口目标责任制考核中连续五年获得一等奖，城市环境综合整治定量考核连续五年保持全省第一，耕地总量连续十四年保持动态平衡。公共安全体系建设扎实推进，安全生产形势持续稳定，探索建立了“三全”维稳体系、“合成化”现代警务体系，市级应急指挥平台建设走在全省前列，我市荣获全国社会治安综合治理优秀市。探索建立了农产品、食品、药品链条式“三品”安全监管模式。“五五”普法依法治理目标任务圆满完成。妇女儿童发展纲要目标基本实现。基层民主政治建设得到加强，建成城市社区91个，农村社区140个，城市社区建设经验被民政部推广。国防动员、民兵预备役建设积极推进，军政军民关系进一步发展，实现了全国双拥模范城“三连冠”。对外交往进一步扩大，已与国内外39个城市缔结友好城市或建立友好合作关系，并与联合国人居署建立了长期合作关系。对台事务、民族宗教、老年人、残疾人、统计、人防、史志、档案、气象、红十字等各项事业都取得了新成果。各项社会事业的进步，既有效提高了全市人民的幸福指数，又进一步展示了全市人民团结进取、奋发有为的精神风采！

　　这是政府自身建设不断加强，行政效能逐步提高的五年。按照市委部署，扎实开展深入学习实践科学发展观、创先争优活动和解放思想大讨论，积极推进法治政府、阳光政府、服务政府和亲民政府建设。自觉坚持依法行政，市政府常务会集体学法形成制度；主动接受人大法律监督、政协民主监督和社会舆论监督，五年累计办理人大代表建议276件、政协委员提案915件，达到了办复率、面办率和满意基本满意率三个百分之百。深入推进政府工作提速提效，相继开展了两轮行政审批制度改革，共精简行政审批事项480项，精简率达到64.1%；新的市级行政审批服务大厅建成启用，网上审批和电子监察系统建成开通，行政审批职能和审批项目“两集中”、行政审批人员和权限“两到位”改革全面推行，审计、监察监督不断加强。建立了政府决策调查摸底、集体研究、专家咨询、社会公示、风险评估、合法性审查、跟踪问效等一整套制度，有效提高了决策的民主化、科学化、法制化水平。建立了政府领导与市民网上对话、接听电话、轮流接访以及行风在线、信息平台等畅通民意与诉求的长效机制。认真履行“一岗双责”，注重源头反腐，严肃查处违法违纪案件，政风行风实现新转变。

　　五年来，我市相继荣获了国家卫生城市、国家环保模范城市、国家园林城市、国家节水型城市、全国绿化模范城市、国家可持续发展先进示范区、中国人居环境奖、联合国人居奖等十几项国家级、国际级荣誉称号。日照的影响力日益扩大，被认可的程度更加提高。在这不平凡的五年里，我们共同见证了日照长高长大、变美变靓。今天的日照，已经成为最具活力和发展潜力的城市之一，成为黄海岸边一座具有鲜明特色的新兴城市。

　　在刚刚过去的2010年，面对国际金融危机的持续冲击和节能减排的巨大压力，我们着力转方式、调结构、保发展、惠民生，圆满完成了年初确定的各项任务目标，实现了经济发展与节能减排的“双主动”、“双确保”。一是坚持在发展中求转变，在调整中谋发展，努力保持经济平稳较快增长。生产总值比上年增长12.5%，地方财政收入增长27.9%。大力开展“大项目建设年”和招商引资活动，全年完成固定资产投资775亿元，比上年增长22.5%，实际到账外资3.49亿美元，一批重点项目建成投产或开工建设，成为新的经济增长点。尽力培育战略性新兴产业和高新技术产业，LED电器、风力发电等一批新兴产业项目落户日照，高新技术产业产值增长40%。认真落实刺激消费的各项政策措施，努力扩大消费需求。全方位开拓国际市场，进出口总值增长47%。二是坚持以破釜沉舟和壮士断臂的决心推进节能减排。对原计划“十二五”淘汰的19条立窑水泥生产线，在2010年7月底前全部淘汰停产。组织实施了市区集中供热改造提升工程，克服工期短、工程量大、地质复杂、环境制约等种种难以想象的困难，圆满实现了预定目标，创下了全国城市集中供热改造一次性施工里程最长、管径最大的纪录，从根本上改变了供热状况，并为今后预留了富余空间，取得了打基础、利长远、节能减排、提高热效、扩大面积、改善空气质量、优化投资环境等多方面的综合效应。在钢铁、热电、热力等企业都采取了相应的实质性措施。积极开展节能减排全民行动，全面推动照明、建筑、电力等重点领域以及社会面上的节能减排。这些措施标本兼治，重在治本，为今后的绿色发展开辟了空间。三是扎实做好“三农”工作。认真组织实施“农业结构调整年”和现代农业“121”科技提升工程，与山农大对接的50个合作项目取得积极进展。粮食总产达到120万吨，创2000年以来的新高；农业结构调整面积达到19.8万亩；植树造林10.7万亩，森林覆盖率达到36.2%。新发展农业龙头企业103家、农民专业合作社511家，建成市级农业标准化生产基地20处。认真做好防汛抗旱、森林防火、重大动物疫病和美国白蛾防控工作。全面落实各项强农惠农政策，共发放各类补贴5.12亿元。加快推进农村住房建设和危房改造，新建农村住房4.36万户，改造危房1.57万户。大力加强农田水利基础设施建设，建成农田水利工程1153处。四是加强城市和港路基础设施建设。完成市级城建投资21亿元，是历史上投入最大的一年。新建改造市区道路14条、污水处理厂8座，新增绿化面积120万平方米。城市管理综合执法水平进一步提高。日照港10个新建泊位实现对外开放；342、335省道改造提升工程竣工通车。五是着力抓好财税金融工作。大力培植骨干财源和新兴财源，优化财政收入结构，税收占财政收入的比例达到83.1%，高于全省4.9个百分点。金融业持续快速发展，支持地方经济发展的力度不断加大。日照银行进一步壮大，继青岛设分行之后又在济南设立分行，综合竞争力跻身全国城商行前五强；兴业银行日照支行开业；成功举办了鲁南临港产业区金融发展论坛暨银企合作洽谈会，签约总金额达252亿元。六是努力保障和改善民生。为750户住房困难家庭发放了经济适用住房补贴，为780户城市最低收入家庭发放了廉租房补贴，在市区配建廉租房200套。医疗、失业、工伤、生育保险全部纳入市级统筹。提高了城乡低保对象和优抚对象抚恤补助标准，新增城乡低保人数2.5万人。启动了社会救助和保障标准与物价上涨挂钩联动机制，向13.5万困难群众发放生活补贴。七是加快发展各项社会事业。完成校舍安全工程投资1.3亿元；在莒县开展了实施国家基本药物制度试点，并在面上展开；继2007年中国水上运动会、第十一届全运会水上项目比赛等重大赛事后，又成功举办了2010年中国水上运动会；第六次人口普查入户登记工作顺利完成；人口自然增长率控制在3.8‰。争创全国双拥模范城“四连冠”通过检查验收。八是扎实开展对口援建工作。高质量、高效率完成了北川援建任务，创造了“日照速度”、“日照质量”，形成了宝贵的“日照援川精神”。对口支援新疆麦盖提县工作扎实有效，与重庆市黔江区结对扶贫协作工作有序展开。

　　各位代表！

　　过去一年及整个“十一五”的发展成就来之不易。这得益于省委、省政府和市委的坚强领导，得益于市人大、市政协和社会各界的监督支持，得益于历届领导班子打下的良好基础，是全市人民团结奋斗的结果。在此，我代表市人民政府，向辛勤工作在各条战线的全市人民，向各级人大代表、政协委员，向各民主党派、工商联、无党派人士、各人民团体，向离退休老领导、老同志，向驻日照人民解放军和武警官兵，向所有关心支持日照建设与发展的社会各界人士和海内外朋友，表示衷心的感谢并致以崇高的敬意！

与省委、省政府的要求和人民群众的期盼相比，我市在经济社会发展中仍存在一些较为突出的矛盾和问题，主要是经济规模不大、实力不强，结构还不够合理，人均GDP水平不高，经济增长的资源环境硬约束加大，科技创新能力不强，社会事业支撑不够，部分群众在就业、增收、上学、就医等方面还存在一些困难，城乡、区域经济社会发展还不平衡，县域经济相对薄弱，软环境有待进一步改善等。对此，我们一定高度重视，采取有力措施，认真加以解决。

　二、“十二五”经济社会发展的总体要求和目标任务

　　“十二五”是我市抢抓蓝色经济区发展机遇，推动科学发展，提升综合实力的关键时期。制定并实施好“十二五”发展规划意义重大。按照中央和省的部署及市委《关于制定日照市国民经济和社会发展第十二个五年规划的建议》的要求，市政府编制了《日照市国民经济和社会发展第十二个五年规划纲要(草案)》。《纲要(草案)》及《说明》已印发大会，请一并审议。

《纲要(草案)》提出的“十二五”时期我市经济社会发展的总体要求是：高举中国特色社会主义伟大旗帜，以邓小平理论和“三个代表”重要思想为指导，深入贯彻落实科学发展观，以科学发展为主题，以加快转变经济发展方式为主线，深入实施“四大战略”、“双轮驱动”，围绕加快建设以日照钢铁精品基地为重点的鲁南临港产业区，打造蓝色经济区，建设我国东部重要的临海产业基地、区域性国际航运物流中心、滨海文化和旅游名城、海洋科技教育创新区，加快构建现代产业体系、民生保障体系、公共安全体系和生态环境体系，推动率先创新优质发展，实现富民强市新跨越，建设海洋特色新兴城市。“十二五”时期，要始终坚持以“五个转变”、“八个调整”为主攻方向，努力推动经济发展方式由突出工业发展向现代工业、现代服务业和现代农业协调发展转变，由高耗能、资源型向清洁、低碳和循环经济发展转变，由投资、消费、出口拉动向消费、投资、出口协调拉动与科技创新强力驱动转变，由陆域经济发展向海陆一体发展转变，由城区、县城发展向城乡统筹、一体化发展转变；着力调高调优全市生产总值占全省的比例、服务业比例、高新技术产业比例、城镇化比例、县域经济比例、科技进步对经济增长的贡献率、轻重工业的比例和城乡居民收入分配比例。

　　“十二五”的主要奋斗目标是：

　　—— 经济实力显著提升。到2015年，全市生产总值达到2000亿元，年均增长12%，人均生产总值达到66000元，超过全省平均水平；地方财政收入突破110亿元，年均增长15%以上。社会消费品零售总额年均增长16%以上，固定资产投资年均增长20%以上，进出口总值达到210亿美元，年均增长12%。

　　—— 经济结构优化升级。现代产业体系基本形成，三次产业比例调整到6﹕56﹕38，战略性新兴产业、海洋产业、文化产业占生产总值的比重分别达到10%、35%、5%。建成冶金、石化、汽车3个主营业务收入过千亿元和浆纸、海洋装备、粮油加工3个主营业务收入过五百亿元的产业集群。港口吞吐量超过3亿吨、260万标箱，打造国家重要枢纽港。

　　—— 城乡统筹协调发展。城镇化水平达到55%，中心城区人口达到110万人。基本完成城市中心区和日照国际海洋城起步区建设，力争日照机场建成通航。城乡基础设施建设一体化和公共服务均等化进程明显加快，新农村建设力争达到全省先进水平。

　　—— 生态环境优美宜居。形成比较完善的生态环境体系，完成省下达的节能减排约束目标，力争有80%的区县、乡镇建成生态县和生态乡镇，争取通过国家生态市验收。

　　—— 社会建设有力加强。民生保障体系更加健全，建立起比较完善的社会保障体系，社会就业比较充分，城镇登记失业率控制在3.5%以内。人口自然增长率控制在5‰以内。社会主义民主法制和精神文明建设全面推进，公共安全体系建设达到更高水平。

　　—— 人民生活更加富足。城镇居民人均可支配收入和农民人均纯收入年均增长12%，低收入者收入明显增加，中等收入群体持续扩大，群众拥有更多的财产性收入，人民生活质量和水平不断提高。

　　各位代表！

　　立足新起点，谋划“十二五”，我们既面临着转方式、调结构，推动率先创新优质发展的新任务，也面临着机遇与挑战交织的新形势，需要审时度势，全面把握，科学务实，积极作为。展望“十二五”的发展，山东半岛蓝色经济区建设已正式上升为国家战略，在“一核、两极、三带、三组团”的规划布局中，以日照钢铁精品基地为重点的鲁南临港产业区被列为两个“增长极”之一，青(岛)潍(坊)日(照)城镇组团被确定为重点打造的“三组团”之一，日照在全国、全省发展大格局中的战略地位空前提高；日照钢铁精品基地等重大生产力布局项目的建设，将为我市临港产业集聚、经济跨越发展提供新的强劲“引擎”；山西中南部铁路大通道、青日连铁路、日照机场等重大基础设施项目的建设，将为我市经济社会发展打造更为有力的支撑条件；鲁南临港产业区区域开放合作省级综合配套改革试点，将为我市加快发展带来充满活力的体制机制环境；国家级日照经济技术开发区、省级经济开发区和高新区、日照保税物流中心、鲁南国际贸易与航运中心等对外开放平台集聚功能不断增强，青岛海湾大桥及隧道开通即将形成青日“一小时经济圈”，将为我市构筑全面开放新格局注入新的动力；新一轮思想解放和“执行力、创新力、公信力”建设年活动深入开展，全市上下干事创业、创先争优的氛围更加浓厚，将为我们加快发展提供坚强有力的保障。只要我们牢固树立和认真落实科学发展观，进一步凝聚起全市上下的智慧和力量，坚定信心，抢抓机遇，开拓进取，真抓实干，就一定能创造更加令人骄傲的业绩，谱写更加辉煌壮丽的篇章！

　　三、2011年的政府工作

　　2011年是实施“十二五”规划的开局之年。综合分析今年面临的形势，既有有利条件，也有不利因素。从国际看，今年世界经济有望实现恢复性增长，但危机并没有结束，甚至可能出现反复。从国内看，我国仍处于重要战略机遇期，中央确定“积极稳健、审慎灵活”的宏观政策取向，实施积极的财政政策和稳健的货币政策，有利于协调经济增长、结构调整与管理通胀预期的关系。但受人民币持续升值、物价上涨等影响，经济发展将面临更多的不确定因素。从我市看，经过“十一五”的发展，全市总体实力跃上新台阶，但总量小、实力不强仍是我市的主要矛盾；主导优势产业已经初露端倪，但集群化程度不高、产业层次偏低的问题仍很突出；城乡面貌显著变化，但二元结构特征仍然明显，城市内涵亟待丰富；民生状况显著改善，但群众生产生活仍然面临诸多困难；我市发展面临的机遇空前增多，特别是打造山东半岛蓝色经济区上升为国家战略，为我市跨越发展提供了千载难逢的重大机遇，但将机遇转化为发展优势仍有大量工作要做。在新的一年里，我们要深入贯彻落实党的十七大和十七届五中全会以及中央、省、市经济工作会议精神，坚持以科学发展为主题，以加快转变经济发展方式为主线，坚持解放思想、变中求进、重点突破，着力增创蓝色经济区域发展新优势，着力保持经济平稳较快发展，着力转型升级、优化结构、提质增效，着力改革创新、扩大开放，着力保障和改善民生，促进全市经济社会率先创新优质发展，确保实现“十二五”良好开局。主要预期目标是：生产总值增长12%，规模以上工业增加值增长16%，固定资产投资增长20%，社会消费品零售总额增长16%，进出口总值增长12%，地方财政收入增长15%，城镇居民人均可支配收入和农民人均纯收入均增长10%，城镇登记失业率控制在3.5%，人口自然增长率控制在5.8‰。着重抓好以下六个方面的工作：

　　(一)坚持增量、存量“双调整”，大力推进转方式、调结构。坚持以增量调整带动存量调整，以存量调整倒逼增量优化，全力做大做优经济“蛋糕”。

　　一是增强投资和消费的拉动作用。继续抓好大项目建设，重点实施好境内总投资近4000亿元的180多个重大项目，力争完成投资270亿元。滚动实施好百项重点工业技改项目，完成投资100亿元以上。进一步完善支持民间投资的政策措施，放宽市场准入，促进社会投资稳定增长和结构优化。建立企业工资正常增长机制，完善最低工资制度，增加城乡居民特别是中低收入群体收入，提高居民消费能力。深入开展“万村千乡市场工程”和“满意消费惠万家”活动，加强“农超对接”和城镇5到8分钟便民消费服务圈建设，着力改善消费条件。认真落实家电下乡和以旧换新等政策，引导汽车、农用车、大型农机具、装饰装修等大宗商品消费，培育新的消费增长点。

　　二是突出发展战略性新兴产业和高新技术产业。加快发展新能源、新信息、新医药、新材料、海洋新兴产业、高端装备制造等战略性新兴产业，启动实施“十二五”期间100个战略性新兴产业项目，年内重点抓好众山生物科技、五征新能源汽车、华润风力发电、海帝电器170万台平板电视等47个总投资102亿元的新兴产业项目，完成投资60亿元。大力实施高新技术自主创新行动计划，鼓励企业与高等院校、科研单位共建技术创新战略联盟和企业技术中心，加快培育一批有较强实力和核心竞争力的创新型企业，确保高新技术产业产值增长30%以上。

　　三是坚定不移地推进节能减排。深入抓好水泥、钢铁、轻工、纺织等重点领域的落后产能淘汰工作，拆除已停产的立窑水泥生产线。积极运用新技术、新工艺推动重点行业结构、能源结构优化升级。加快推进节能减排重点工程建设，新建、扩建第三污水处理厂、香店河污水处理厂、碧波废水处理中心等5座污水处理厂以及多个生活区污水处理站。继续抓好全社会节能减排，大力推广节能照明，新建节能建筑200万平方米，完成市区50万平方米既有建筑节能改造。全面抓好工程、管理和结构减排。积极用好财税杠杆，实现节能减排与发展绿色经济、培植地方新兴财源的“双突破”，确保完成省政府下达的节能减排任务目标。

　　四是切实加强资源集约节约利用和环境保护。严格执行投资项目用地、节能、环保、安全等准入标准，切实提高投资效益。坚持实施最严格的耕地保护和水资源总量控制制度，积极开展农村土地综合整治和城乡建设用地挖潜，实行用水限额管理制度，高效利用好每一寸地、每一滴水。规范和加强再生资源市场管理，大力发展再生资源回收利用和精深加工。加强对农村、工业园区、水源地、海岸带、山体等重点区域的生态保护，全面开展流域环境、空气环境综合整治，确保顺利通过国家环保模范城市复查，保护提升环境质量。

　　(二)坚持农业基础地位不动摇，确保农业增产农民增收。认真贯彻落实中央农村工作会议“大兴水利强基础，狠抓生产保供给，力促增收惠民生，着眼统筹添活力”的总要求，以更加有力的措施抓好“三农”工作。

　　一是毫不放松地抓好农业生产和结构调整。突出抓好“米袋子”、“菜篮子”生产，坚持粮食生产“三条底线”不动摇，大力推广良种良法配套技术，突出抓好10万亩小麦高产创建示范片，不断提高粮食综合生产能力，确保粮食安全。加快推进精品蔬菜基地建设，新发展设施大棚蔬菜1.5万亩。加快发展特色农业，年内新发展茶园3万亩，以承办全国茶叶学会年会暨第九届“中茶杯”全国名优茶评比颁奖活动为契机，进一步做好提升品质、整合资源、打造品牌等工作，推动我市茶产业迈上新台阶。提升水产业，壮大畜牧业，发展林果业，年内发展浅海生态健康立体养殖1万亩，规模化畜禽养殖场100个，特色经济林3万亩，新增造林面积10万亩。大力实施产业化经营、标准化生产、品牌化营销、科技化提升战略，集中力量扶持一批农业龙头企业和标准示范园区，规范发展农民专业合作社，提高农业组织化、产业化水平。

　　二是狠抓水利基础设施建设。牢固树立大水利观念，立足长期抗旱，提高水资源保障能力，全面完成潮白河、巨峰河综合治理及50座小型水库除险加固，争取开工建设马陵水库、户部岭水库除险加固工程，做好五莲龙潭沟水库、“西水东调”一期工程前期工作。在全省率先启动小型水利工程建设，采取“拦、蓄、引、集”等多种措施，规划建设一批投资少、见效快的小塘坝、小水池等集蓄工程，逐步解决农村“当家水”的问题。去年9月份以来，我市出现300年一遇的特大干旱，目前旱情仍在持续，必须切实抓好抗旱工作。要加强对水资源的统一管理和科学调度，把保证城乡居民生活用水放在首要位置，统筹解决好城市与农村、工业与农业、生产与生活用水，大力开展节约用水工作，把旱灾造成的损失降到最低限度。

　　三是加快推进新型城镇化步伐。在充分尊重农民意愿、切实保障农民利益的前提下，按照“政府主导、规划先行、合村并居、综合配套、村企村区结合、政策性与市场化结合”的机制和思路，积极稳妥地推进新型农村社区建设。继续改善农村公共服务设施和农民生产生活条件，新建农村住房2.2万户，改造农村危房8500户，进一步扩大轻钢结构新型农房试点；继续实施农村饮水安全工程，解决90个村、7万人的饮水安全问题；改造农村公路150公里；新建农村综合服务中心20处。

　　四是不折不扣地落实中央和省、市强农惠农扶农政策。按照中央“三个重点、三个确保”的要求，继续加大“三农”投入，做到财政支出重点向农业农村倾斜，确保总量和增量均有提高；预算内固定资产投资重点用于农业农村基础设施建设，确保总量和比重进一步提高；土地出让收益重点投向农业土地开发、农田水利和农村基础设施建设，确保足额提取、定向使用。抓好农村信用社改革和小额贷款公司、村镇银行试点工作，引导金融机构加大涉农信贷投放力度。严格落实良种、农机具购置等各项扶持政策，确保各项补贴按时发放到农民手中。

　　(三)抢抓蓝色机遇，壮大特色经济。紧紧抓住山东半岛蓝色经济区上升为国家战略的机遇，加快培育具有海洋特色和比较优势的现代产业体系。

一是提升鲁南临港产业区承载能力。按照“一带三轴八园一城”的空间布局，统筹配置资源要素、统筹建设基础设施、统筹布局产业项目、统筹落实扶持政策，进一步完善重点园区功能，形成多点支撑、多极发展的新格局。年内完成日照国际海洋城概念性总体规划、重点地区及起步区概念性城市设计和产业发展规划编制工作，对6平方公里的起步区进行基础设施配套，努力建设海洋产业发达、服务功能完善、生态环境优美的海洋新城。加快推进鲁南临港产业区区域开放合作省级综合配套改革试点，积极做好设立日照保税港区、探索中日韩自由贸易区先行试点的争取工作，为发展蓝色经济、打造优势产业构建优质载体和优越的体制机制环境。

　　二是培植壮大临港产业集群。坚持把日照钢铁精品基地建设作为“头号工程”，扎实做好重组和配合服务、配套产业规划等工作，力争早日开工建设。进一步加强与现代起亚等汽车企业的合作，加快推进现代派沃泰40万台汽车自动变速箱、龙泰集团30万台微型乘用车、岱摩斯6万台车桥、五征集团10万辆微卡等项目，全力打造新兴汽车产业基地。依托岚山港区30万吨级原油码头和三条石油长输管线，努力打造化工产业基地。加快推进亚太森博浆纸三期、森达美120万吨棕榈油深加工等项目建设，努力打造环保循环型浆纸制造和粮油加工产业基地。切实抓好18个重点产业集群培育，搞好临港大工业与县域、镇域经济对接，促进区域经济统筹协调和城乡一体化发展。

三是推动服务业跨越发展。依托港口、航贸中心和保税物流中心，加快发展港口物流业。抓好旅游集散中心、海洋嘉年华、阳光海岸等重点项目建设，创新奥林匹克水上公园管理体制和运行机制，提高开放和综合利用程度，推动旅游与体育、文化等融合发展。着力打造四季游、休闲度假游和高档精品游，力争游客接待量达到2300万人次，实现旅游总收入140亿元。加大对电子商务企业整顿规范力度，推进“电子商务示范城”建设。积极引进和培育物联网示范企业，争创国家级物联网创新示范园区。大力引进国内外知名金融机构和后台服务系统，积极推动日照银行跨区域经营，努力打造地方精品银行，加快建设区域性金融服务中心。培育发展国际贸易、市场交易、中介咨询、财务、法律等商务服务业，建立完善现代商务服务体系。

　　(四)全面扩大对外开放，积极推进各项改革。坚持以我为主招商选资，进一步加大产业招商、专业招商、园区招商、以商招商和资本招商力度，突出对战略性新兴产业、高新技术产业、先进制造业和现代服务业等低耗能、低排放或低耗能、无排放项目的招引，原则上带烟囱冒黑烟的项目一律不要，决不以牺牲环境为代价追求一时的发展。积极开展国内外招商推介活动，对已签约项目实行档案化管理和跟踪制落实，切实提高项目履约率和资金到位率。加快转变外贸发展方式，大力实施“科技兴贸”和“品牌带动”战略，提高机电和高新技术产品出口比重，培育自主知识产权和自有品牌企业，培植建设汽车及零部件、生物医药、海洋化工、电子信息等出口创新基地。支持企业“走出去”，打造境外加工贸易基地、能源资源开发基地，带动国产设备、原材料和零部件出口以及重要资源、关键技术设备进口。深入实施“东吸亚太、西连桥带、北接半岛、南引长珠”的区域发展战略，加强与鲁南临港产业带、陆桥沿线地区的协作，精心组织好陆桥沿线部分城市第九次联谊活动；积极推进与长三角、珠三角、京津唐等经济圈的联系，切实提高我市的区域竞争力。以青岛跨海大桥和海底隧道开通为契机，进一步深化与青岛的全面合作，努力实现同城化发展。扎实做好对口支援新疆麦盖提县和结对帮扶重庆黔江区工作。

　　以更大的决心和力度推进各项改革。深化公共财政改革，完善转移支付制度，提高县乡财政保障能力。稳妥推进水电油气等资源性产品价格改革，落实阶梯式水价电价政策。积极推进乡镇机构改革、集体林权制度改革，稳妥推进农村土地承包经营权流转，发展多种形式的适度规模经营。扎实推进医药卫生体制改革，在政府举办的基层医疗卫生机构全面实施国家基本药物制度。加快金禾生化、众山生物、双港电子、遨游汽车配件等企业上市步伐，并培育一批后备上市资源。

　　(五)抓好城市和港路基础设施建设，提升载体服务功能。按照“东港、南工、北游、西新、中城”的功能布局，坚持高起点规划、大手笔建设、精细化管理，加快建设海洋特色新兴城市。

　　 一是倾力打造城市中心区和阳光海岸等精品工程。按照“高档、高层、精品”的原则，加快推进城市中心区建设，全面启动拆迁工作，力争用两到三年的时间基本形成规模，努力打造能够满足国际商务、旅游、休闲、体育等需求，体现日照特色的高档中央商务区和富有特色的活力街区。实施阳光海岸整治续建工程，把提升环境与引导消费结合起来，对万平口至水芙蓉段按城市滨海旅游岸线标准进行精心建设，对水芙蓉至森林公园段按郊野生态岸线标准进行生态恢复，建成具有国际水准的海滨风景带和旅游消费带。

　　二是加强城市规划建设和管理。坚持以科学的规划引领城市建设，年内完成城市总体规划修编，编制日照北部区域近期建设详细规划。投入市级城建资金22亿元，实施市级综合文化中心、香店河治理和滨河生态特色街、东关北路商业街、植物园改造等城市重点工程，新增绿化面积126万平方米，加快建设北海路等18条城市道路，启动18个城中村的拆迁改造。对老城区、城乡结合部、旧住宅区以及小街小巷进行环境综合整治。按照精细化、科学化、人性化的要求，进一步提高城市管理效能。积极推进城市数字化管理，启动数字城市地理信息空间框架和数字城管信息平台建设，加快各类数字信息平台的统筹利用和资源共享。

　　三是大力推进重大基础设施建设。年内投资30亿元，着力抓好岚山港区30万吨级矿石码头、石臼港区焦炭码头等10项港口重点工程，力争启动新的30万吨级原油码头建设，努力使港口货物吞吐量达到2.39亿吨、130万标箱。建成日照港疏港高速、长深高速日照段、岚山港疏港路、日照至东明原油管道一期工程，加快山西中南部铁路大通道、坪岚铁路扩能改造、胶新铁路电气化改造、日照至仪征输油管道建设，推进青日连沿海铁路、日照机场、日潍高速、枣临岚高速、日照至淄博输油管线和204、206国道日照段升级改造等项目。加快启动莒县、五莲县驻地到市区的快速通道建设，构建“半小时交通圈”。建成秦楼、稍坡220千伏和汪家台、南湖110千伏输变电工程。

　　(六)深入推进民生保障体系建设，积极发展社会事业。落实促进就业的各项政策措施，多渠道开辟就业门路，重点抓好高校毕业生、农村转移劳动力、城镇就业困难人员等群体就业，鼓励自谋职业和自主创业，年内新增城镇就业2.5万人，转移农村劳动力1.6万人。落实工资集体协商制度，保障劳动者权益。加快构建覆盖城乡居民的社会保障体系，扎实做好新农保工作，积极推进城镇居民养老保险制度建设，城市低保标准提高到每人每月320元，农村提高到每人每年1400元。落实好“五线一网”医疗救助办法，逐步提高新农合和城镇居民医保补助、住院费用报销比例，进一步解决好关闭破产企业退休人员和困难企业职工的参保问题。完善住房保障体系，开工建设公共租赁住房。继续采取综合措施，有效稳定物价总水平。

　　全面落实《中长期教育改革和发展规划纲要(2010—2020年)》及我市《实施意见》，推动各类教育协调发展。深入实施素质教育，加强乡镇中心幼儿园建设，推动中小学标准化建设和校舍安全工程，推进义务教育均衡发展和普通高中教育优质发展，打造职业技能培训基地，提高职业教育基础能力，启动高等职业教育统筹发展试点。认真落实《中长期人才发展规划纲要(2010—2020年)》，加大人才培养和引进力度，为加快发展提供有力的人才智力支撑。认真落实《关于促进文化产业振兴的意见》，加快培育壮大文化创意、新闻服务、演艺娱乐、影视制作、动漫游戏等十大文化产业，进一步健全完善基层公共文化服务体系，推动两城遗址公园保护规划建设工作，加快建设滨海文化名城。大力实施“健康日照行动”，加强基层医疗卫生设施建设，全面做好疫病防控工作，巩固提高国家卫生城市创建成果。精心筹办2011年中日韩国际帆船赛等重要文体活动，充分利用游泳馆、奥林匹克水上公园等体育设施，大力开展全民健身运动，提升“水上运动之都”品牌。严格落实人口目标管理责任制，稳定低生育水平，提高出生人口素质。继续做好“双拥共建”和优抚安置工作，加强国防动员、民兵预备役建设，实现全国双拥模范城“四连冠”。出台并认真落实《公共安全体系建设规划》，加强社会治安综合治理，深入开展严打整治斗争，大力推进治安防控体系建设，进一步增强人民群众的安全感。健全社会稳定风险评估机制，努力从源头上预防和减少矛盾。强化安全生产监督管理和食品、药品、农产品安全监管以及应急救援能力，加快人防指挥体系建设。启动实施“六五”普法和依法治市规划，完善法律援助和法律服务。依法搞好村委会换届选举工作。

　　四、加强政府自身建设

　　面对“十二五”和今年经济社会发展的繁重任务，各级政府及工作人员要更加注重加强自身建设，按照市委的部署，深入开展新一轮思想解放和“三力建设年”活动，努力打造法治政府、阳光政府、服务政府、亲民政府。

　　一是着力提高各级政府的执行力、创新力、公信力。牢固树立“雷厉风行、崇尚行动、追求一流、重在结果”的执行理念，进一步转变作风，真抓实干，对决策的事情形成说干就干、干就干好的风气，切实提高政府执行力。大力推进工作创新，着力转变不适应不符合科学发展的思想观念和行为方式，努力形成以创新促发展的新局面。深入贯彻落实《政府信息公开条例》，不断完善政务公开、办事公开、新闻发布等制度，坚持言必信、行必果，不折不扣地兑现落实各项政策和承诺，切实提高各级政府的公信力。

二是坚持执政为民。树立正确的政绩观，多办顺民意、解民忧、增民利的实事，多做谋长远、打基础、利全局的工作，努力以促发展、惠民生的实际成效赢得群众信任。充分发挥市长公开电话、市长信箱、网上对话、行风在线、短信平台的作用，进一步拓宽民意诉求渠道，及时解决群众反映的合理诉求和困难问题。高度关注下岗职工、老弱病残、鳏寡孤独、未就业毕业生及城乡低保对象等弱势群体，积极帮助他们解决就业再就业、生活保障、子女上学、看病等方面的问题。精心组织实施2011年度为民办实事重点项目，既抓普惠性的大工程，又抓区域性、方便群众、关系民生的小项目，让全市人民共享改革发展成果。

　　三是自觉依法行政。严格按照法定权限和程序行使权力、履行职责，自觉接受市人大及其常委会的法律监督和工作监督，接受政协和各民主党派的民主监督以及人民群众和社会、舆论的监督，切实做好人大代表建议、政协委员提案办理工作。健全科学民主的决策机制，建立重大决策责任追究制度，做到决策与责任相统一。全面深化“两集中、两到位”和公共资源交易市场化改革，推进依法阳光运作，进一步简化办事程序，推动政府工作提速提质提效。严格行政问责和责任追究，坚决纠正不作为、乱作为和慢作为，对由此导致不良影响的要发现一起处理一起，下大决心进一步优化发展环境。

　　四是严格廉洁从政。认真贯彻落实《廉政准则》“8个禁止、52个不准”的要求，切实抓好反腐倡廉制度建设，不断完善建设工程招投标、经营性土地招拍挂、政府采购和“收支两条线”等制度，实行“阳光财政”，建立廉政建设的长效机制。强化监察、审计专门监督，做到行政权力运行到哪里、监督就跟踪到哪里，财政资金运用到哪里、审计就跟进到哪里。坚持勤俭办一切事业，大力压缩一般性开支。认真落实“一岗双责”，切实把好自身廉洁关、分管部门干部队伍的防腐关、家人与身边工作人员的清廉关，老老实实做人，踏踏实实做事，虚心接受监督，以清正廉洁的实际行动，赢得全市人民的信赖与支持，努力打造廉洁勤政、务实高效、一心为民的政府！

　　各位代表！

　　“十一五”的辉煌已载入史册，“十二五”的征程已经开启。面对千载难逢的重大机遇，站在蓄势待发的历史节点，让我们更加紧密地团结在以胡锦涛同志为总书记的党中央周围，高举中国特色社会主义伟大旗帜，在市委的坚强领导下，万众一心，开拓奋进，努力开创我市科学发展、跨越发展、和谐发展的新局面，以优异成绩向建党90周年献礼！
